# Effect of Drinking Water Salt Content on the Interaction between Surfactants and Bacteria

**DOI:** 10.1128/spectrum.01011-23

**Published:** 2023-07-06

**Authors:** Nimrod Shteindel, Alon Silberbush, Yoram Gerchman

**Affiliations:** a Department of Evolutionary and Environmental Biology, University of Haifa, Haifa, Israel; b Oranim College of Education, Tivon, Israel; c The Institute of Evolution, University of Haifa, Haifa, Israel; JMI Laboratories

**Keywords:** *Pseudomonas aeruginosa*, biofilms, cleaning, detergents, disinfectants, water

## Abstract

Sodium dodecyl sulfate (SDS) is a common surfactant used in various hygienic products. Its interactions with bacteria were studied previously, but the three-way interaction between surfactants, bacteria, and dissolved salts in the context of bacterial adhesion has not been studied. Here, we examined the combined effects of SDS (at concentrations typical of everyday hygienic activities) and salts, sodium chloride, and calcium chloride (at concentrations typically found in tap water) on the adhesion behavior of the common opportunistic pathogen Pseudomonas aeruginosa. We found that bacterial adhesion in the absence of SDS was dependent on the cation concentration rather than the total ionic strength and that combined treatment with several millimolar NaCl and SDS can increase bacterial adhesion. The addition of low concentrations of SDS (2 mM) to tens to hundreds millimolar concentrations of NaCl, typical of systems that suffer seawater incursion, reduced bacterial adhesion dramatically. Combined treatment with Ca^+2^ (in concentrations typical of those found in hard water) and SDS produced a small increase in total adhesion but a dramatic increase in the strength of adhesion. We conclude that the type and concentration of salts in water can have a considerable effect on the efficacy of soap in reducing bacterial adhesion and should be taken under consideration in critical applications.

**IMPORTANCE** Surface-adhering bacteria are a reoccurring problem in many settings, including households, municipal water systems, food production facilities, and hospitals. Surfactants, and specifically sodium dodecyl sulfate (also known as SDS/SLS), are commonly used to remove bacterial contamination, but data regarding the interaction of SDS with bacteria and especially the effects of water-dissolved salts on this interaction are lacking. Here, we show that calcium and sodium ions can dramatically affect the efficacy of SDS on bacterial adhesion behavior and conclude that salt concentrations and ion species in the water supply should be considered in SDS applications.

## INTRODUCTION

Surfactants are a cornerstone of modern hygiene, facilitating the removal of bacteria from surfaces (1, 2). Many works have studied the effects of surfactants on the removal of mature biofilms from surfaces (3). Other studies have delved into the interactions between surfactants and hydrophobic compounds and the parameters controlling the formation of emulsions, allowing the removal of hydrophobic substances from surfaces—temperature, ionic strength, and the specific structure of the surfactant (4). Here, we address the effects of surfactants and salts on the very first stages of biofilm formation adherence. Bacteria are much more complex than simple hydrophobic compounds, making their interactions with surfactants complex. They are not emulsified by surfactants—bacterial surface charges are typically negative, making them hydrophilic. Bacteria are also orders of magnitude larger than micelles, the nanometer-scale spherical structures formed by surfactants in emulsions. In contrast to hydrophobic substances, bacteria cannot be contained in the micelles. Indeed, studies on surfactant adsorption suggest that surfactants work by adding or neutralizing charges on the bacterial surface, changing its electrostatic interactions with the surface to which it adheres (5). Nonetheless, the formation of micelles is important in this regard, as they allow surfactants to inhabit the bulk liquid, increasing bacterial interaction with them.

Both bacteria and surfactants interact with dissolved salts, affecting their behavior. The critical micelle concentration (CMC), the threshold concentration of surfactant required for the formation of micelles, is inversely correlated with the ionic strength of the solution (4). Higher salt concentrations facilitate the formation of micelles, allowing a higher portion of the surfactant to migrate to the bulk liquid and interact with bacteria. Bacterial adhesion is also dependent on ion concentrations, as described by the DLVO model (6–8), developed to describe the formation of colloids but applied to bacterial adhesion (i.e., reference 9). This model analyzes the effects of charge in the water on the interactions between submerged surfaces. Charged particles in the water can mask charges on the bacterial surface, reducing electrostatic repulsion and allowing surface adhesion through van der Waals interactions, working over shorter distances. Higher ion concentrations form a double layer of charges, causing the reassertion of electrostatic repulsion. Optimal bacterial adhesion has been demonstrated in median ion concentrations by several studies (9, 10).

While surfactants facilitate the removal of bacteria from surfaces (see references 11–13), the effects of dissolved salts on this interaction are not well studied. Studies of the interactions between bacteria and surfactants used constant salt concentrations (14–16), but since both bacterial adhesion and surfactant activities are affected by concentration in a nonlinear manner, their combined effects should be studied.

Here, we study the effects of SDS and salts on the initial adhesion kinetics of the model organism Pseudomonas aeruginosa, an opportunistic pathogen that can cause disease in plants and animals, including humans, known for its stickiness and surface-bound lifestyle (17). We measured the effects of salts and SDS on bacterial adhesion at a time resolution of seconds/minutes in a high-throughput setting (10), allowing us to test many surfactant and salt concentrations in combination. Sodium dodecyl sulfate and salt concentrations were chosen to reflect real life conditions, to gain insights relevant to everyday hygienic practices.

## RESULTS

### SDS effect on bacterial adhesion.

**FIG 1:**
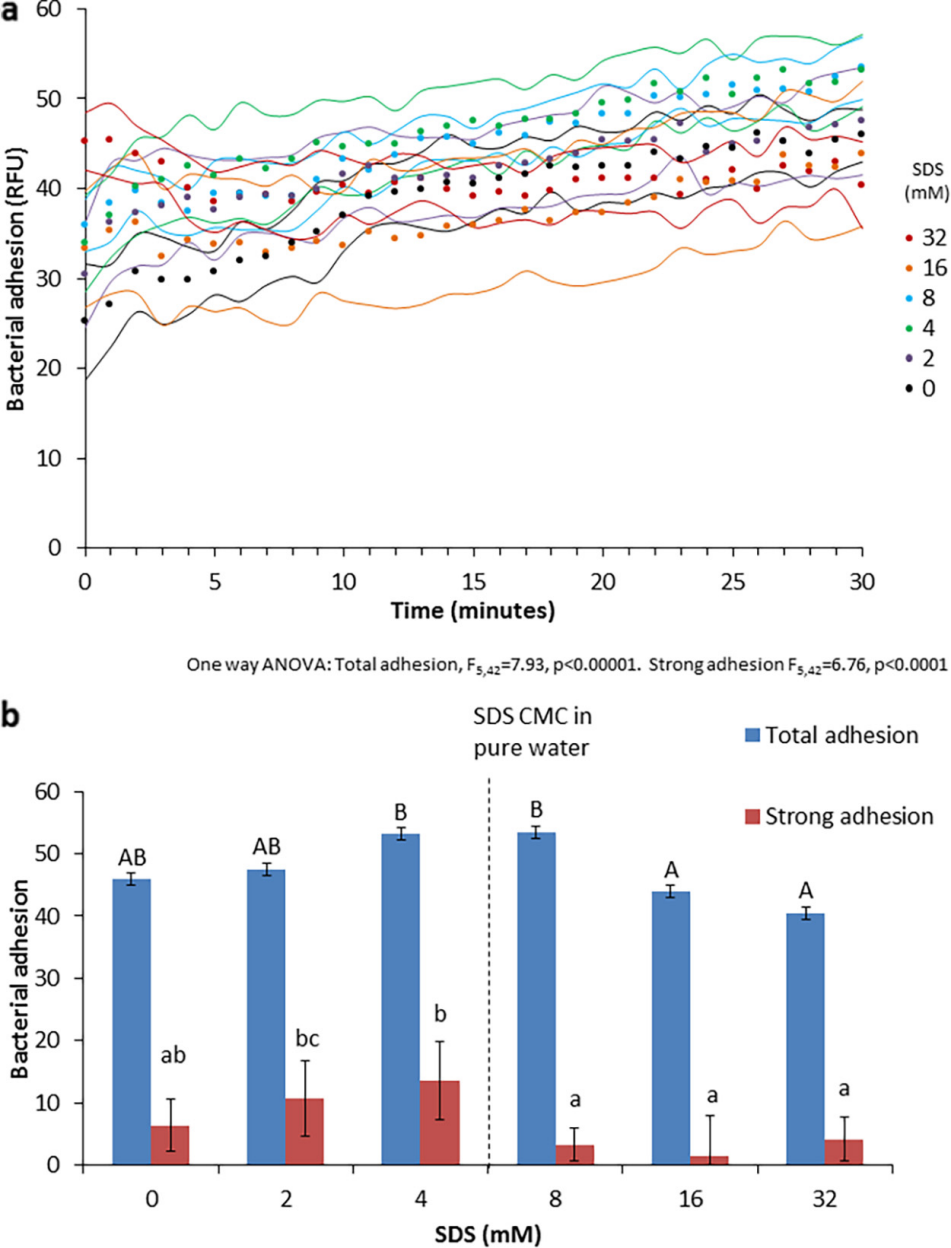
Effect of SDS concentration on P. aeruginosa PAO1 adhesion. (a) Kinetic measurements of bacterial adhesion. Dots indicate the measurement times; the flanking curves represent ±1 SD (*n* = 8 per treatment). (b) Total adhesion at 30 min and strong adhesion after weakly adhering bacteria were removed by pipetting (*n* = 8). The error bars represent ±1 SD. The capital letters above the total adhesion bars indicate statistically significantly different groups (*P* < 0.05). The lowercase letters above the strong adhesion bars represent statistically significantly different groups (*P* < 0.05 using one-way ANOVA).

Sodium dodecyl sulfate had a small but statistically significant effect on P. aeruginosa adhesion in deionized water (Fig. 1). Concentrations up to the CMC caused an increase in adhesion, correlated with the SDS concentration. The trend reversed at higher concentrations, with a notable decrease from the peak adhesion seen in 4 and 8 mM SDS (Fig. 1b). Adhesion strength (bacteria that were not washed away when liquid was removed by pipetting) displayed a similar pattern (Fig. 1b).

### Sodium chloride and calcium chloride effect on bacterial adhesion.

**FIG 2:**
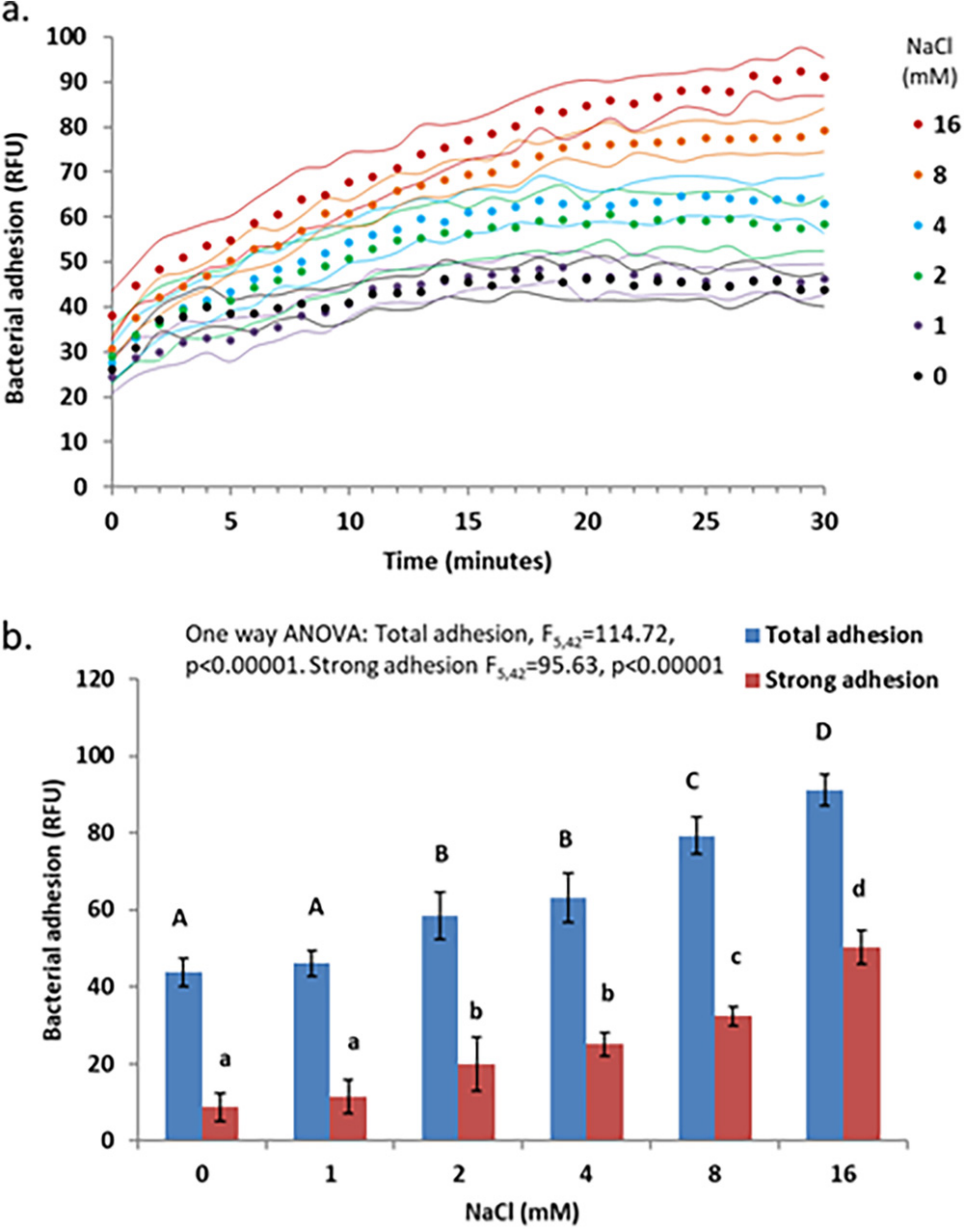
Effect of NaCl concentration on P. aeruginosa PAO1 adhesion. (a) Kinetic measurements of bacterial adhesion. Dots indicate the measurement times; the flanking curves represent ±1 SD (*n* = 8 per treatment). (b) Total adhesion at 30 min and strong adhesion after weakly adhering bacteria were removed by pipetting (*n* = 8). The error bars represent ±1 SD. The capital letters above the total adhesion bars indicate statistically significantly different groups (*P* < 0.05). The lowercase letters above the strong adhesion bars represent statistically significantly different groups (*P* < 0.05).

**FIG 3:**
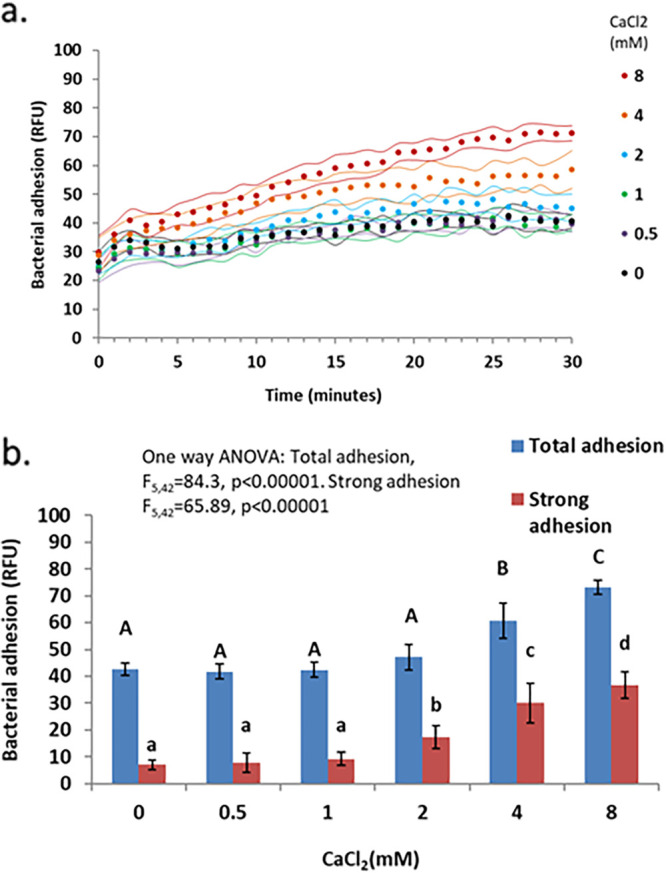
Effect of CaCl_2_ concentration on P. aeruginosa PAO1 adhesion. (a) Kinetic measurements of bacterial adhesion. Dots indicate the measurement times; the flanking curves represent ±1 SD (*n* = 8 per treatment). (b) Total adhesion at 30 min and strong adhesion after weakly adhering bacteria were removed by pipetting (*n* = 8). The error bars represent ±1 SD. The capital letters above the total adhesion bars indicate statistically significantly different groups (*P* < 0.05). The lowercase letters above the strong adhesion bars represent statistically significantly different groups (*P* < 0.05).

The addition of either NaCl (Fig. 2) or CaCl_2_ (Fig. 3) in concentrations typical of those in drinking water caused a dose-dependent increase in adhesion and adhesion strength.

**FIG 4:**
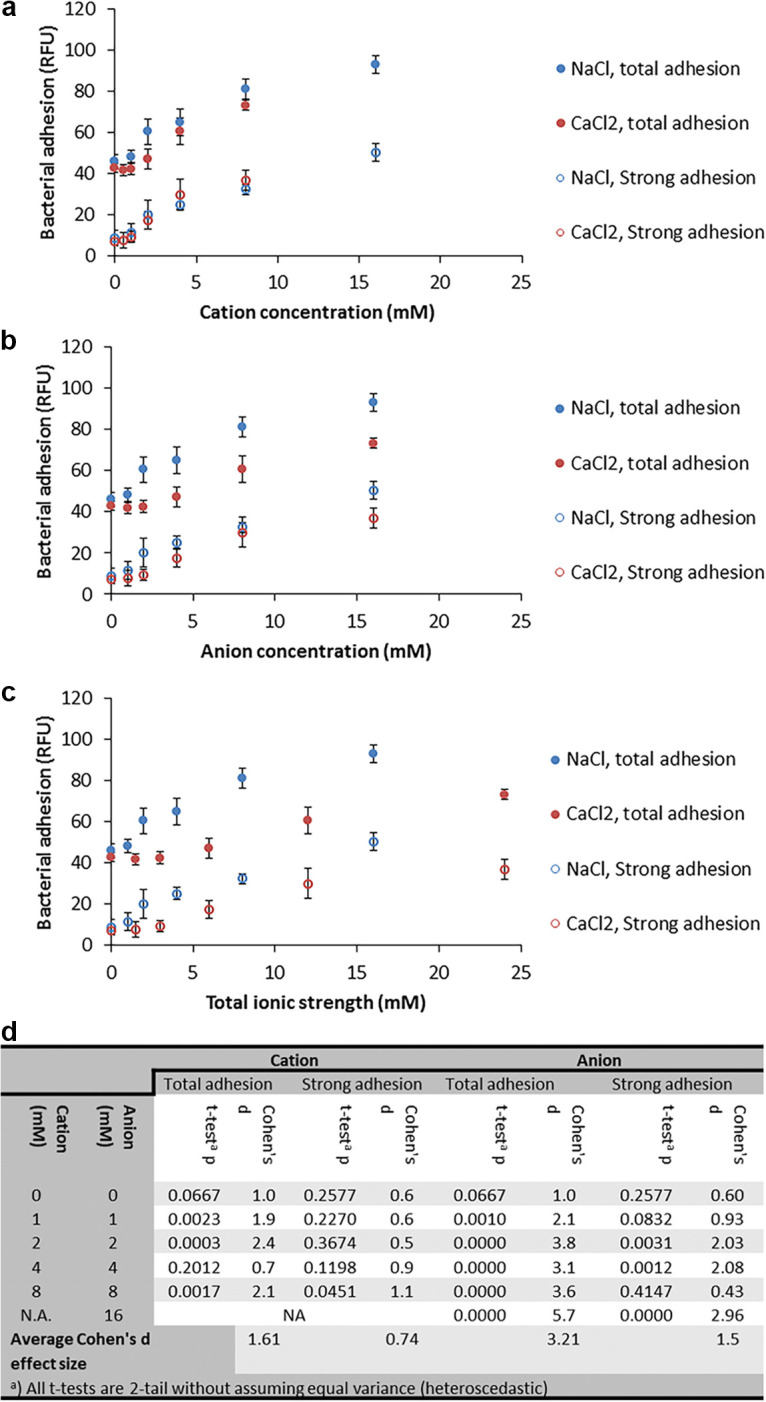
Effect of NaCl or CaCl_2_ concentration on P. aeruginosa PAO1 total adhesion at 30 min (alternative representations of the data presented in Fig. 2 and 3). (a) Adhesion versus cation (Na^+^ or Ca^+2^) concentration; (b) adhesion versus anion (Cl^−^) concentration; (c) adhesion versus ionic strength. The closed circles show total adhesion after 30 min; strong adhesion (open circles) was measured after loosely adhering bacteria were removed by pipetting (*n* = 8 for all measurements). The error bars represent ±1 SD. (d) Statistical comparison of adhesion at similar cation/anion concentration. The *t* test was used to establish statistically significant differences and Cohen’s D size effect to estimate the size of differences.

Figure 4 shows alternative representations of the data presented in Fig. 2 and 3, using the same adhesion data (with salts and no SDS) but charting it against the cation concentration, anion concentration, and total ionic strength. Adhesion and strong adhesion were similar in parallel cation concentrations, regardless of the cation species (Fig. 4a) or anion concentration, and although differences were found to be statistically significant, they were small (as indicated by Cohen’s D effect size analysis; Fig. 4d). Conversely, when adherence was charted against the anion concentration (Fig. 4b) or against the total ionic strength (Fig. 4c), very different values were seen under similar salt concentrations (Fig. 4d). These results suggest that adhesion responds to cation concentration and not to anion concentration or total ionic strength.

### Interaction effects of salts and SDS.

**FIG 5:**
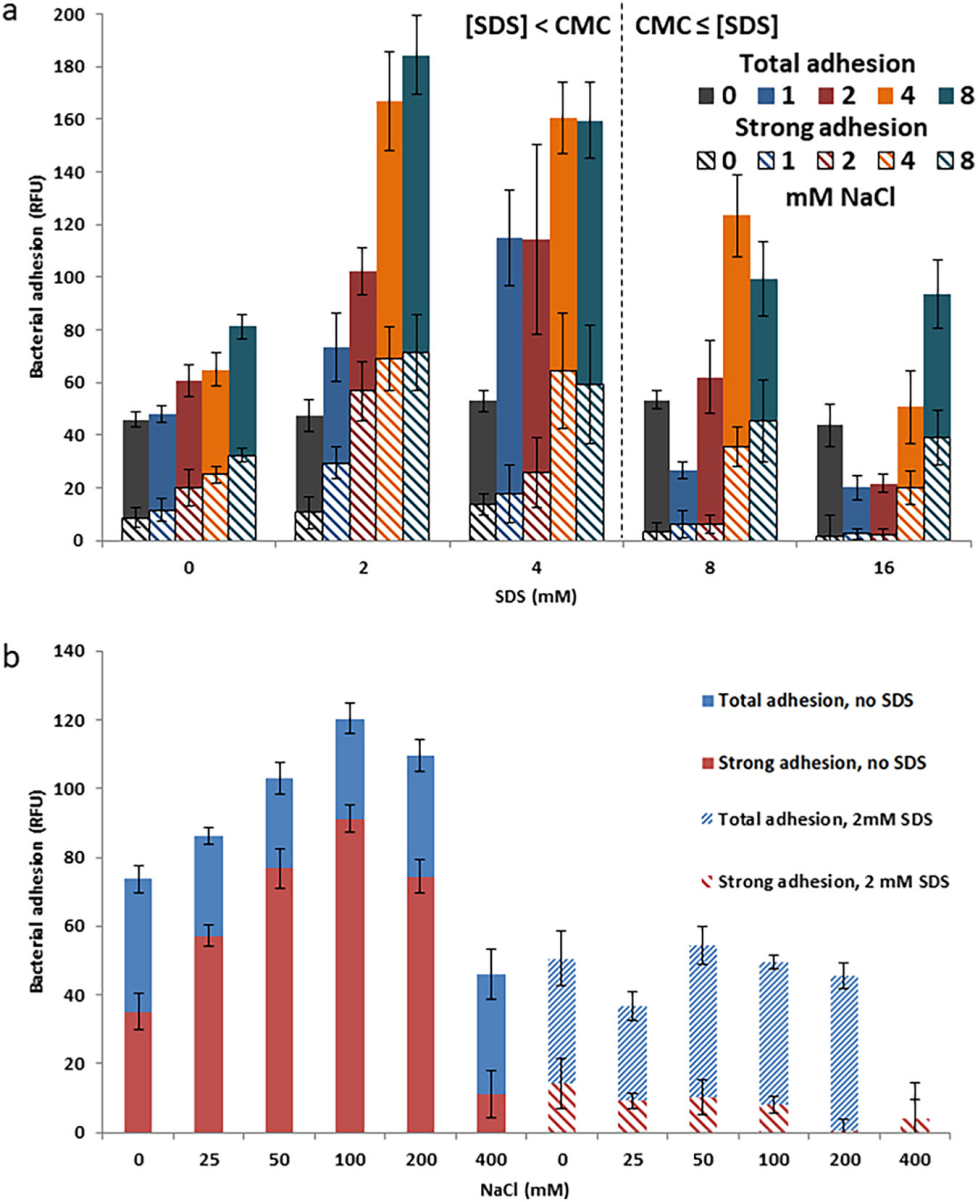
Effect of combined NaCl and SDS treatments on P. aeruginosa PAO1 adhesion. (a) Adhesion at 30 min; the solid bars show total adhesion at 30 min, and the striped bars show strongly adhering bacteria (*n* = 6 per treatment). The error bars represent ±1 SD. (b) Adhesion at higher salt concentrations with or without 2 mM SDS (*n* = 7 per treatment). The error bars represent ±1 SD.

Combined treatment with NaCl and SDS produced complex results (Fig. 5). Sodium dodecyl sulfate concentrations lower than 8 mM enhanced the effect of NaCl, increasing the total bacterial adhesion (Fig. 5a, left) while reducing strong adhesion. Sodium dodecyl sulfate concentrations greater than 8 mM (Fig. 5a, right) with 4 and 8 mM NaCl caused higher adhesion, while lower concentrations (1 mM NaCl with 8 mM SDS; 1 and 2 mM with 16 mM SDS) reduced it (compared to adhesion with the same NaCl concentrations in the absence of SDS). Higher salt concentrations (Fig. 5b) increased adhesion and adhesion strength, up to a concentration of 100 mM NaCl, and reduced adhesion at higher concentrations. Addition of 2 mM SDS reduced adhesion and adhesion strength to the levels seen in pure water and suppressed adhesion strength altogether across all concentrations.

**FIG 6:**
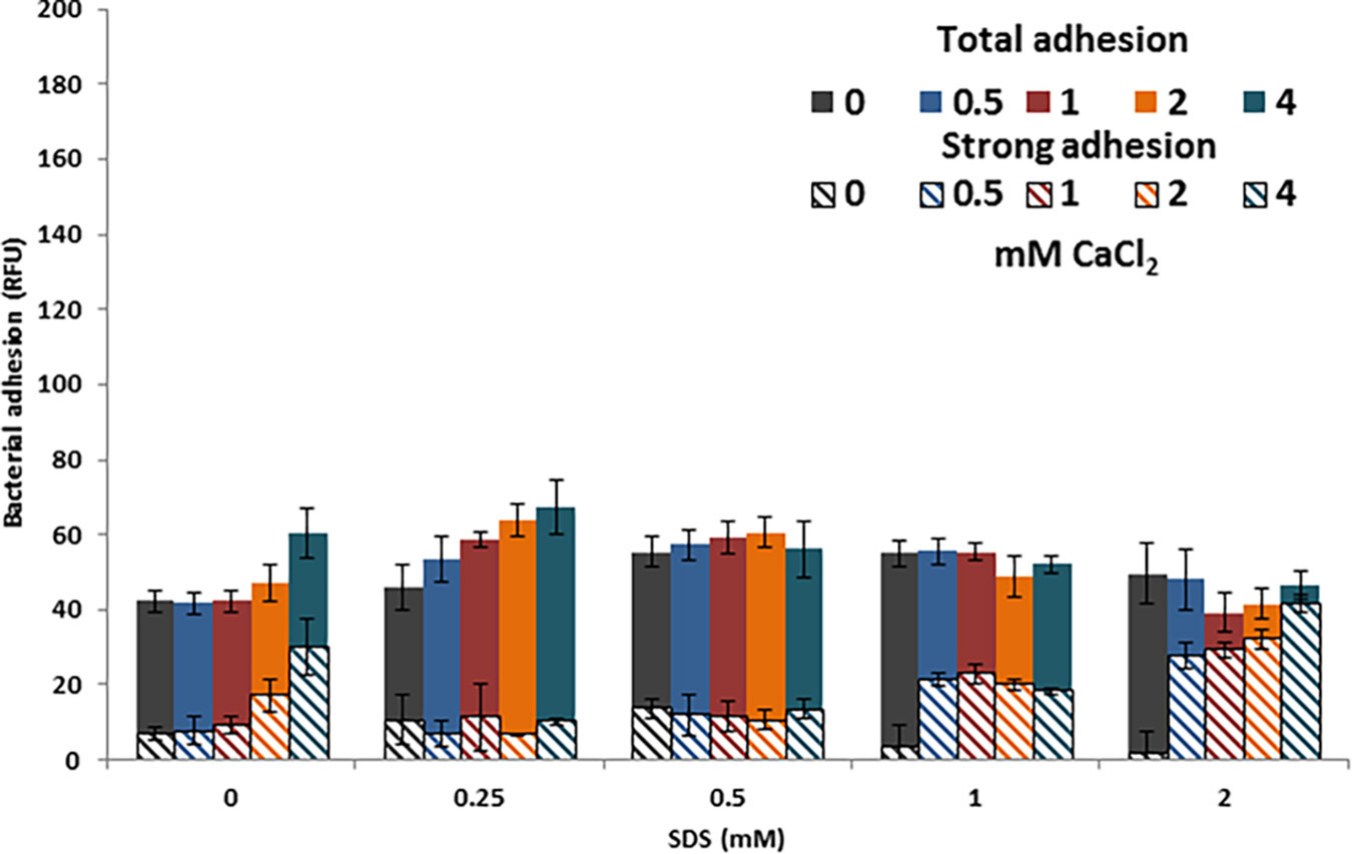
Effect of CaCl_2_ and SDS on total and strong adhesion of P. aeruginosa PAO1 cells at 30 min (*n* = 7). The error bars represent ±1 SD.

Combined treatment with CaCl_2_ and SDS had a milder effect on total adhesion compared to NaCl-SDS treatment (Fig. 6). The addition of CaCl_2_ to SDS in concentrations of 8 and 16 mM caused an increase in strong adhesion, correlated with both the CaCl_2_ and SDS concentration.

## DISCUSSION

Soaps and surfactants are in common use and have been studied rigorously for their physicochemical characteristics (4, 18, 19). Nevertheless, the three-way interactions between surfactants, salts, and bacteria, in the context of bacterial adhesion behavior, are not well understood. Previously published research looking into these interactions used buffers at concentrations affecting both the surfactant CMC and bacterial adhesion (10 to 150 mM) (14–16), making interpretation complex. NaCl and CaCl_2_ were used here at concentrations representing those in realistic water systems, with the calcium ion concentration limited by its precipitation as Ca(dodecyl sulfate)_2_ at 8 mM SDS (seen as a floating film; see also reference 18).

Here, we show the existence of three-way interactions between SDS, salts, and bacteria over a wide range of surfactant and salt concentrations. The addition of only SDS at sub-CMC concentrations improved adhesion in a dose-dependent manner, probably as a result of the complementary Na^+^ ion introduced with the SDS. At sub-CMC concentrations, the dodecyl sulfate (DS) ions were mostly concentrated at the surface and thus not interacting with the bacteria in the bulk liquid, while the counter sodium ions were able to disperse throughout the volume and interact with a far larger portion of the bacterial population. The difference in adhesion at these SDS concentrations versus the comparable NaCl concentrations (53 ± 4.02 RFU in 4 mM SDS versus 63 ± 6.44 RFU at 4 mM NaCl; Fig. 1b and 2b, respectively) may result from some heterogeneity in the sodium concentrations arising from the attraction of some of the Na^+^ ions to the DS ions at the liquid surface, preventing them from interacting with the bacteria in the bulk liquid. When SDS concentrations exceeded the CMC, micelles were formed, and the DS concentration in the bulk liquid increased, allowing it to interact with bacteria and adsorb onto their surface via the hydrophobic tail, increasing the bacterial negative charge and reducing adhesion (5).

NaCl increased adhesion and adhesion strength in a dose-dependent manner up to 100 mM, while reducing both at higher NaCl concentrations. CaCl_2_ yielded a similar pattern, with a smaller increase in adhesion and adhesion strength. These results are in line with the DLVO theory describing the effects of ionic strength on colloid formation, often used in modeling bacterial adhesion processes (9, 20). The adhesion substratum used in this work was tissue culture (TC)-treated polystyrene, a polar surface, which holds a partial negative charge due to the presence of oxygen-containing functional groups such as hydroxyl and carboxyl (21). Bacterial surface charge tends to be negative, causing bacteria to be repulsed from such surfaces (9). This repulsion could be masked by cations (such as Na^+^ and Ca^+2^) in concentrations typical of those in drinking water, thus increasing adhesion. At higher concentrations, the Na^+^ and Ca^+2^ cations can form layers on both the surface and the bacterial surface, reestablishing electrostatic repulsion and reducing adhesion. Indeed, the correlation between adhesion and the cation concentration, rather than ionic strength, supports this hypothesis and suggests that the DLVO model, which uses ionic strength as a predictor of adhesion (9), should be updated.

In combined treatment with NaCl and SDS concentrations below 8 mM, SDS caused an increase in total adhesion that can be attributed to the sodium ions introduced with the surfactant (SDS being a salt of Na^+^ and [dodecyl sulfate]^−^). This treatment also caused a reduction in adhesion strength. Higher SDS concentrations (8 and 16 mM) combined with 1 and 2 mM NaCl reduced adhesion and adhesive strength. In municipal water systems, these effects may translate into different adhesion patterns, depending on the salt content of the water. In developed countries, NaCl concentrations in drinking water are generally limited to 4 mM, as higher concentrations affect the flavor of the water (https://www.epa.gov/sdwa/drinking-water-regulations-and-contaminants; accessed 16 June 2022). Under these conditions, dilution of SDS products below the CMC is likely to cause some excess adhesion. In contrast, in tap water in regions suffering from seawater incursion into costal aquifers, NaCl concentrations can reach as high as 140 mM (22). These conditions promote bacterial adhesion, but a low SDS concentration will reduce adhesion dramatically. This increase in SDS efficacy at reducing bacterial adhesion at high NaCl concentrations is due to the influence of ionic strength on the SDS CMC—25 mM NaCl reduces the CMC of SDS from 8 mM to ~3 mM, while 100 mM NaCl reduces it to ~1.5 mM (4), allowing the surfactant to interact with the bacteria at far lower concentrations.

Combined treatment with CaCl_2_ and SDS produced a small effect on total adhesion but significantly increased the fraction of strongly adhering bacteria, in correlation with both the SDS and CaCl_2_ concentrations. Dissolved calcium in drinking water originates in calcium-rich sediments or rocks and can reach up to 220 mg/L (quantified as dissolved CaCO_3_), corresponding to 2.2 mM Ca^+2^ (23). These calcium concentrations increase the strength of bacterial adhesion. An increase in bacterial adhesion strength at higher calcium ion concentrations was previously reported (24) and explained by specific interactions with bacterial adhesins on the bacterial surface. This explanation suggests that the effect is specific to certain bacterial species. Calcium ions may form a bridge between negative charges on the plate surface and bacterial surface generated by the dodecyl sulfate coating, producing a similar effect. As SDS (or DS) adsorption onto bacteria seems to be a ubiquitous phenomenon, the effect of SDS on bacterial adhesion strength in the presence of calcium may occur with all bacteria, regardless of species. No similar effect is produced by sodium ions, most probably due to their smaller charge and Van der Waals radius.

In applications where prevention of bacterial adhesion is essential, such as food processing facilities or clinical setting (hospital, outpatient facility, etc.), cation species in water should be considered and adjusted, if needed. In water with a low sodium content—below 8 mM—the use of SDS at sub-CMC concentrations is likely to increase bacterial adhesion, making diluted soap solutions counterproductive, as the decrease in bacterial adhesion will occur only at higher SDS concentrations. This scenario is likely in developed countries and inland regions. In areas suffering from seawater incursion into costal aquifers, bacterial adhesion is likely to be fast and strong, but the addition of low SDS concentrations (~0.6 g/L) should reduce it dramatically. In karstic regions, where high dissolved Ca^+2^ concentrations are expected, the addition of SDS may increase the strength of bacterial adhesion. Higher SDS concentrations could reverse this effect, but Ca^+2^ concentrations higher than 8 mM might result in removal of the surfactant due to precipitation, requiring a water softening treatment for efficient prevention of bacterial adherence.

## MATERIALS AND METHODS

### Strain, plasmid, media, and cultivation conditions.

Pseudomonas aeruginosa PAO1 wild type (WT) was obtained from the Manoil laboratory at Washington University (25). Cells were transformed with pMRP9-1 constitutively expressing carbenicillin resistance and GFP_mut2_ fluorescence protein (26, 27) and used throughout this work. Bacteria were streaked from freezer stock onto LB agar and inoculated into homemade M9 medium (47.8 mM Na_2_HPO_4_, 22 mM KH_2_PO_4_, 8.6 mM NaCl, 18.7 mM NH_4_Cl, 2 mM MgSO_4_, 0.1 mM CaCl_2_, 0.4% glucose; all from Sigma, Israel) supplemented with 200 μg/mL carbenicillin (Sigma). The bacteria were cultivated in 6 mL medium in 18-mm glass tubes placed in a water bath shaker set to 37°C and 120 rpm for 18 h. The bacteria were collected by centrifugation (12,000 × *g*, 2 min), washed once, and resuspended in deionized water.

### Preparation of NaCl, CaCl_2_, SDS, and Allura red AC stock solutions.

All chemicals were purchased dry from Sigma and dissolved in sterile deionized water to make the following stock solutions: 32 mM NaCl, 16 mM CaCl_2_ (2H_2_O), 64 mM SDS (equivalent to 8 CMC in deionized water), and 100 mg/mL Allura red AC.

### Microtiter plates.

The microtiter plates were clear, flat-bottom, 96-well plates made of sterile TC-treated polystyrene (SPL, South Korea).

### Preparation of 96-well plate and adherence experiments.

**FIG 7:**
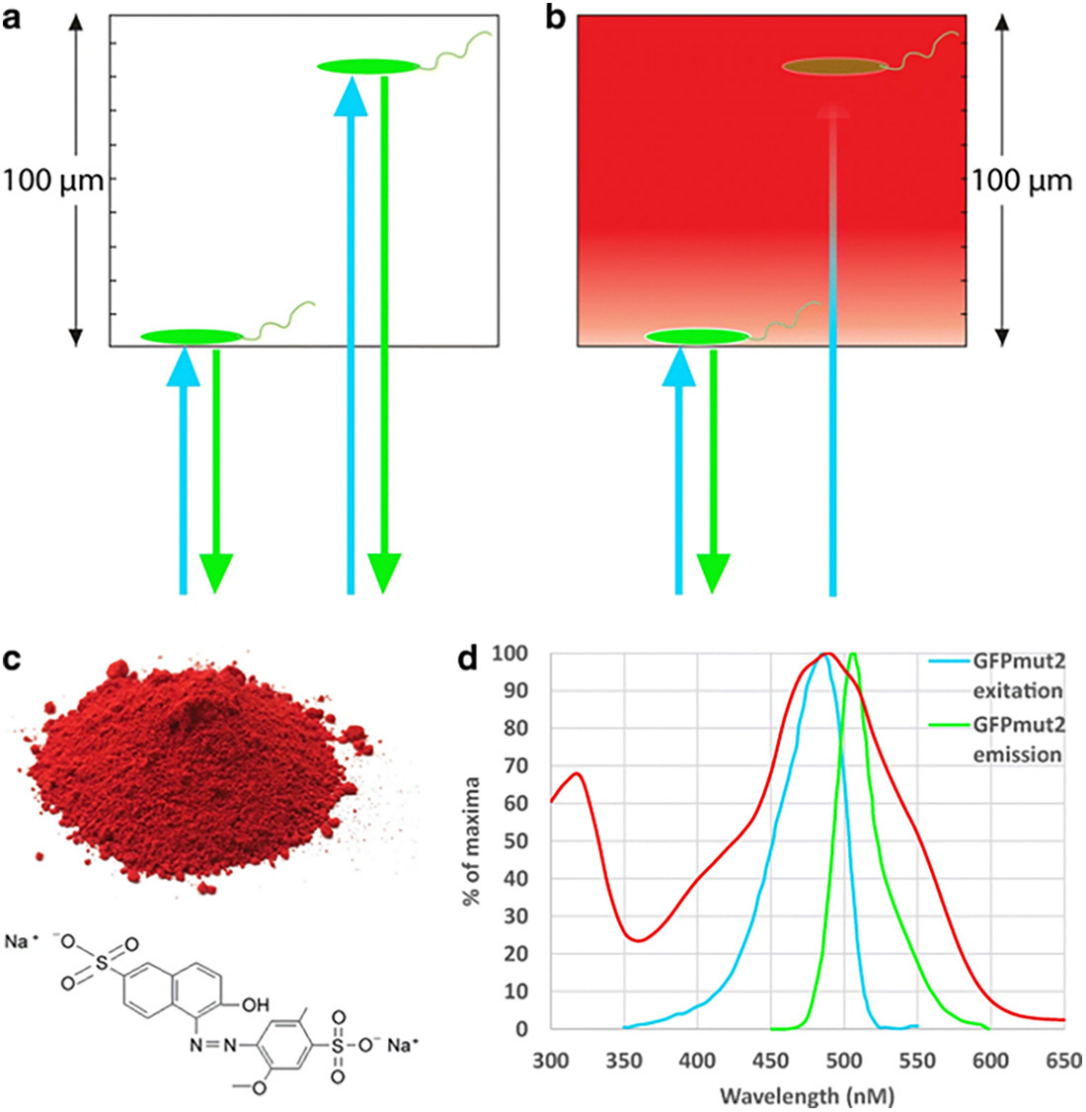
(a and b) Fluorescence of adhering and nonadhering bacteria within a microtiter well in the absence (a) and presence (b) of dye. (c) Allura red AC powder and molecule structure. (d) Allura red AC absorption spectrum versus GFP_mut2_ excitation and emission spectra. (Figure adapted from reference 10.)

The effect of salts and SDS on bacterial adhesion kinetics was studied using the method described in reference 9 (Fig. 7). Briefly, a suspension of GFP-expressing bacteria was added to the wells of a 96-well plate containing a solution of dye that absorbed light in the excitation and emission wavelengths of GFP, masking the fluorescence from planktonic bacteria (10). Where designated, 50 μL double concentration solutions of SDS, salts, or their mixtures were preadded to the wells. Just before starting a kinetic measurement, 50 μL bacterial suspension at an optical density at 600 nm (OD_600_) of 0.1 in deionized water (double working concentration; predetermined in 100 μL volume in a 96-well plate) supplemented with 1.6 mg/mL Allura red AC was added to each well, diluting the bacterial culture density to an OD_600_ value of 0.05, dye to 0.8 mg/mL, and salt/SDS to the designated concentrations. Bacteria were added using a multichannel pipettor, taking less than 30 s from the first to last column of the plate. In all cases, the plates were loaded immediately into the plate reader (Synergy HT; Biotek, VT, USA) and read for bottom fluorescence (excitation, 485/20 nm; emission, 528/20 nm; gain, 60) once a minute for 30 min. After the last kinetic point was measured, the liquid was removed, along with sedimented, nonadhering, and weakly adhering bacteria, using a multichannel pipettor, and the wells were filled with 100 μL of 0.8 mg/mL Allura red AC solution in deionized water. The wells were read once more for bottom fluorescence, measuring the fraction of bacteria tightly bound to the well bottom. Eight replicates were performed for each experiment unless otherwise noted.

### Relative fluorescence units.

The intensity of fluorescent signals is highly dependent on the specific fluorometer used. Thus, the results are reported in relative fluorescent units (RFU).

### Statistical analysis.

Statistical analyses were performed using SPSS statistics version 24.0.0 (IBM; 2016); Tukey’s honestly significant difference (HSD) test was used as a *post hoc* test in all cases.

### Data availability.

All data are presented in the text; the raw data are available by request from the corresponding author.
